# Self-Perception of Aging and Hypertension in a Cohort of Sexual Minority

**DOI:** 10.7759/cureus.43127

**Published:** 2023-08-08

**Authors:** Alan P Jacobsen, Brittanny M Polanka, Deanna Ware, Sabina A Haberlen, Mark Brennan-Ing, Steven Meanley, Chukwuemeka N Okafor, Frank J Palella, Robert K Bolan, M. Reuel Friedman, Michael Plankey

**Affiliations:** 1 Department of Medicine, Johns Hopkins Medical Institutions, Baltimore, USA; 2 Division of Epidemiology and Community, University of Minnesota School of Public Health, Minneapolis, USA; 3 Department of Medicine, Georgetown University Medical Center, Washington, DC, USA; 4 Department of Epidemiology, Bloomberg School of Public Health, Johns Hopkins University, Baltimore, USA; 5 Department of Geriatrics, Brookdale Center for Healthy Aging, City University of New York, New York, USA; 6 Department of Family and Community Health, University of Pennsylvania School of Nursing, Philadelphia, USA; 7 Department of Public Health, Robbins College of Health and Human Sciences, Baylor University, Waco, USA; 8 Department of Medicine, Northwestern University Feinberg School of Medicine, Chicago, USA; 9 Department of Family Medicine, Los Angeles LGBT Center, Los Angeles, USA; 10 Department of Urban-Global Public Health, Rutgers, the State University of New Jersey, Rutgers, USA

**Keywords:** sexual minority men, multicenter aids cohort study, hiv, self-perception of aging, hypertension

## Abstract

Objectives

To determine whether self-perception of aging is an important marker of health and hypertension among older sexual minority men.

Methods

We evaluated associations between self-perception of aging (chronologic-subjective age discrepancy and aging satisfaction) and hypertension among 1,180 sexual minority men (51.6% with HIV/48.4% without HIV) from the Multicenter AIDS Cohort Study using a manifest Markov chain model adjusted for HIV status, age, race/ethnicity, education, smoking status, inhaled nitrite use, diabetes, dyslipidemia, kidney and liver disease.

Results

The overall prevalence of hypertension increased from 73.1% to 82.6% over three years of follow-up. Older age discrepancy (aOR (adjusted odds ratio): 1.13 95% CI: 0.35-3.69) and low aging satisfaction (aOR: 0.88; 95% CI: 0.31-2.52) were not associated with an increased prevalence of hypertension, regardless of HIV status.

Discussion

More than 80% of sexual minority men had a diagnosis of hypertension but self-perception of aging was not predictive of incident hypertension.

## Introduction

Sexual minority adults are disproportionately affected by psychosocial (e.g., discrimination, psychiatric illness), behavioral (e.g., tobacco use and poor diet), and physiological (e.g., inflammation and autonomic nervous system reactivity) stressors which contribute to cardiovascular risk factors such as hypertension, diabetes, hyperlipidemia, and obesity which drive cardiovascular morbidity and mortality [1]. Although earlier studies reported no consistent signal of increased hypertension prevalence specifically among sexual minority men (SMM) [2], national representative studies have found that gay and bisexual men had higher odds of hypertension than those who identified as heterosexual [3,4]. For example, an analysis of the National Longitudinal Study of Adolescent Health Study found that SMM had a prevalence of hypertension of 38.7% as compared with a prevalence of 27.2% in heterosexual men [5].

An individual’s self-perception of aging (SPA) is one such underexplored psychosocial stressor which may contribute to hypertension and cardiovascular disease. SPA is composed of assessments of subjective age and aging satisfaction, which are indicators of positive adaptation to age-related losses, age identity, and successful aging [6,7]. Subjective age is defined as how old an individual perceives themself to be [8]. Aging satisfaction captures overall subjective well-being in terms of usefulness and vitality at their age [9]. SPA has been shown to be associated with cardiovascular reactivity, i.e., acute increases in blood pressure and heart rate in response to stress [10], but its association with hypertension in the absence of a stressor is unclear. Although the most recent analysis of the Health and Retirement Study found significantly higher rates of hypertension among those with a higher subjective age, an earlier wave had found no association between blood pressure and subjective age [11,12]. Similarly, in the English Longitudinal Study of Ageing, those who reported feeling younger than their chronological age were less likely to report a diagnosis of hypertension [13], whereas in another study, older individuals who were exposed to negative aging stereotypes demonstrated an increase in blood pressure when exposed to a mathematical or verbal challenge [10].

By studying participants in the Multicenter AIDS Cohort Study (MACS), a prospective cohort of SMM with or without HIV, we aimed to determine whether negative SPA was independently associated with hypertension. We also hypothesized that this association would be greater for people living with HIV (PLWH) compared to those living without HIV (PLWOH), due to increased risks of aging-related co-morbidities imposed by HIV [14].

## Materials and methods

Study design, setting, and participants

The Multicenter AIDS Cohort Study (MACS) is a prospective cohort study of SMM living with or without HIV in four sites in the United States: Baltimore/Washington, DC; Chicago, IL; Los Angeles, CA; and Pittsburgh, PA/Columbus, OH. MACS participants attend semiannual visits that collect social, behavioral, and medical history data and biospecimens through an Audio Computer-Assisted Self-Interview and standardized clinical examinations. The study design of the MACS has been described elsewhere [15].

Conducted over six semiannual visits between April 2016 and March 2019, the Understanding the Patterns of Healthy Aging Study is a sub-study of the MACS that seeks to understand psychosocial resiliency factors that contribute to healthy aging among older SMM with and without HIV. To be eligible, participants must have been at least 40 years old on April 2016, reported at least one incidence of sexual intercourse with another man since enrolling in the MACS, and completed at least one in-person MACS in the two years prior to enrollment in April 2016 [16]. There were 1,180 participants included in the analytic sample. We obtained data for these participants at visits 62 (October 2014-March 2015), 63 (April 2015-September 2015), 64 (October 2015-March 2016), and 70 (October 2018-March 2019).

Measures and procedures

Outcome

Hypertension (HTN) was defined as either a measured systolic blood pressure ≥ 130 mm Hg or diastolic blood pressure ≥ 80 mm Hg, a prior diagnosis of hypertension, or the use of anti-hypertensive medication [17]. Blood pressure was measured at visits 64 and 70. Blood pressure was measured from a bare arm, with an appropriately sized cuff, in a seated position after resting for 5 minutes. A second recording was made after waiting 5 minutes and the mean of the two measures was recorded.

Primary Predictors

Age discrepancy and aging satisfaction were assessed at visits 62 or 63 (whenever data was first available). Age discrepancy was calculated as the difference between subjective age (“What age (years) do you feel most of the time?”) and chronological age. Age discrepancy was calculated as the difference between the age a participant “feels” most of the time and their chronological age [18]. It was categorized into three strata: feeling older, feeling the same, and feeling younger than their chronological age for the model. Aging satisfaction was assessed using the Attitudes Towards Aging subscale from the validated Philadelphia Geriatric Center Morale Scale [19,20]. The Cronbach’s alpha in this sample is -0.56. The subscale included five items: 1) “Things keep getting worse as I get older (Yes/No)”; 2) “I have as much pep as I had over the past 6 months (Yes/No)”; 3) “As I get older, I am less useful (Yes/No)”; 4) “I am as happy now as I was when I was younger (Yes/No)”; and 5) “As I get older, things are __ than I thought they would be (Better/Worse).” “Yes” and “Better” responses were assigned a value of 2 and “No” and “Worse” responses were assigned a value of 1. Items 1 and 3 were reverse-coded. All items were summed to obtain a score that ranged from 5 to 10. The resulting values were then categorized into low (5-6), moderate (7), and high (8-10) aging satisfaction.

Covariates

Participants’ chronological age at each visit was calculated from self-reported date of birth and date of visit. Race was categorized as non-Hispanic white, non-Hispanic Black, Hispanic, and other race. Education was categorized as less than some college and some college or higher. HIV status (PLWH/PLWOH) was assessed using enzyme-linked immunosorbent assay with confirmatory Western blot on all MACS participants at their initial visit and at every visit for PLWOH at the previous visit. PLWH included all participants that were identified as such at their initial visit and those who seroconverted during study observation. Substance use covariates included self-reported stimulant use (yes/no), marijuana use (yes/no), erectile dysfunction drug use (yes/no), inhaled nitrite use (yes/no), alcohol consumption (none/low-moderate/moderate-heavy/binge) and smoking status (never/former/current). Comorbidities included diabetes (fasting glucose ≥ 126 mg/dL), liver disease (serum glutamic pyruvic transaminase or serum glutamic oxaloacetic transaminase > 150 U/L), kidney disease (estimated glomerular filtration rate < 60 mL/min/1.73 m^2^ or urine protein-to-creatinine ratio ≥ 200), and dyslipidemia (total cholesterol ≥ 200 mg/dL or low-density lipoprotein cholesterol ≥ 130 mg/dL or high-density lipoprotein cholesterol < 40 mg/dL or triglycerides ≥ 150 mg/dL) [21]. Weight status was measured by body mass index (BMI, kg/m^2^; underweight/normal weight/overweight/obese). Liver and kidney disease were collapsed into a single dichotomous variable (liver or kidney disease/no liver or kidney disease). Participants were classified as having a hepatitis C infection if they seroconverted or had an acute infection or chronic infection. Time invariant covariates such as HIV status, race/ethnicity, and education were obtained at visit 62 or 63. Time variant covariates such as age, substance use, and comorbidities were measured at visits 64 and 70.

Statistical analysis

Descriptive statistics on the primary independent variables, covariates, and outcomes were summarized by HIV status. We used a manifest Markov chain model to study the change over time in observed variables [22]. We generated the probability of hypertension status changes (transition probabilities), which is defined as the probability of hypertension status (HTN/no HTN) at visit 70, given the blood pressure status at visit 64. There are four mutually exclusive transition patterns: 1) no HTN to no HTN; 2) no HTN to HTN; 3) HTN to no HTN; and 4) HTN to HTN. Individuals who transitioned from HTN to no HTN were considered to still have a clinical diagnosis of HTN given the absorbing nature of the condition, i.e., a patient’s blood pressure can be controlled, even without medications, but they retain the diagnosis. Transition probabilities represent the probability of HTN status at visit 70, given their HTN status at visit 64. A multinomial logistic regression model was used to test the association of aging discrepancy and aging satisfaction on HTN transition patterns, adjusting for covariates reported at visits 64 and 70. The final adjusted model included the primary predictors (age discrepancy and aging satisfaction), HIV status, age, race/ethnicity, education, smoking status, inhaled nitrite use, diabetes, dyslipidemia, and kidney/liver disease. Data were analyzed using MPLUS version 8.4 (Muthén & Muthén, Los Angeles, CA, USA) and SAS version 9.4 (SAS Institute Inc., Cary, NC, USA).

Missing data

There was complete data for the transition patterns for all 1,180 participants. However, there were missing data for some of the covariates. As a result, only 864 participants were included in the multinomial logistic regression modeling.

## Results

Participant characteristics and hypertension prevalence

Among the 1,180 (51.6% PLWH/48.4% PLWOH) participants included in the analytic sample, the median age at visit 64 was 61 years (IQR: 56.0-66.0). Participants were mostly non-Hispanic white (72.7%) and had a college education (60.7%). The prevalence of hypertension was 73.1% and 82.6% at visits 64 and 70, while antihypertensive use at each visit was 44.5% and 50.0%, respectively. Among PLWH, 94.4% were taking antiretroviral therapy at visit 64 and this was not significantly different at visit 70. Additional baseline demographic and clinical data by HIV status are presented in Table 1.

**Table 1:** Population Characteristics, Perception of Aging and Hypertension by HIV Diagnosis (N = 1180) BMI, Body mass index; HTN, Hypertension; IQR, Interquartile range; PLWOH, People living without HIV; PLWH, People living with HIV.

	PLWOH	PLWH	Overall	p-value
N (%)	609 (51.6)	571 (48.4)	1180	
Age at visit 64 (years), Median (IQR)	63.0 (58.0, 69.0)	59.0 (55.0, 64.0)	61.0 (56.0, 66.0)	<0.0001
Aging Satisfaction, Median (IQR)	8.0 (8.0, 9.0)	8.0 (7.0, 9.0)	8.0 (7.0, 9.0)	0.2586
Aging Discrepancy, Median (IQR)	-13.0 (-19.0, -5.0)	-11.0 (-18.0, -1.0)	-12.0 (-19.0, -2.0)	0.0044
HTN at Visit 64, n (%)				
No	170 (27.9)	145 (25.4)	315 (26.7)	0.0568
Yes	438 (71.9)	425 (74.4)	863 (73.1)	
Missing	1 (0.2)	1 (0.2)	2 (0.2)	
HTN at Visit 70, n (%)				
No	154 (25.3)	158 (27.7)	312 (26.4)	0.2762
Yes	455 (74.7)	411 (72.0)	866 (73.4)	
Missing	0 (0.0)	2 (0.4)	2 (0.2)	
Race, n (%)				
Non-Hispanic White	483 (79.3)	375 (65.7)	858 (72.7)	<0.0001
Non-Hispanic Black	94 (15.4)	140 (24.5)	234 (19.8)	
Hispanic	22 (3.6)	49 (8.6)	71 (6.0)	
Other	10 (1.6)	7 (1.2)	17 (1.4)	
Education, n (%)				
Less than College	203 (33.3)	261 (45.7)	464 (39.3)	<0.0001
Some College or Higher	406 (66.7)	310 (54.3)	716 (60.7)	
Stimulant Use at Visit 64, n (%)				
Yes	33 (5.4)	57 (10.0)	90 (7.6)	0.0021
No	550 (90.3)	477 (83.5)	1027 (87.0)	
Missing	26 (4.3)	37 (6.5)	63 (5.3)	
Stimulant Use at Visit 70, n (%)				
Yes	28 (4.6)	61 (10.7)	89 (7.5)	<0.0001
No	553 (90.8)	476 (83.4)	1029 (87.2)	
Missing	28 (4.6)	34 (6.0)	62 (5.3)	
Marijuana Use at Visit 64, n (%)				
Yes	134 (22.0)	156 (27.3)	290 (24.6)	0.0177
No	449 (73.7)	378 (66.2)	827 (70.1)	
Missing	26 (4.3)	37 (6.5)	63 (5.3)	
Marijuana Use at Visit 70, n (%)				
Yes	156 (25.6)	186 (32.6)	342 (29.0)	0.0047
No	423 (69.5)	349 (61.1)	772 (65.4)	
Missing	30 (4.9)	36 (6.3)	66 (5.6)	
Erectile Dysfunction Drug Use at Visit 64, n (%)				
Yes	35 (5.7)	55 (9.6)	90 (7.6)	0.0084
No	548 (90.0)	479 (83.9)	1027 (87.0)	
Missing	26 (4.3)	37 (6.5)	63 (5.3)	
Erectile Dysfunction Drug Use at Visit 70, n (%)				
Yes	43 (7.1)	49 (8.6)	92 (7.8)	0.2987
No	537 (88.2)	488 (85.5)	1025 (86.9)	
Missing	29 (4.8)	34 (6.0)	63 (5.3)	
Inhaled Nitrite Use at Visit 64, n (%)				
Yes	111 (18.2)	138 (24.2)	249 (21.1)	0.0064
No	472 (77.5)	396 (69.4)	868 (73.6)	
Missing	26 (4.3)	37 (6.5)	63 (5.3)	
Inhaled Nitrite Use at Visit 70, n (%)				
Yes	101 (16.6)	132 (23.1)	233 (19.7)	0.0032
No	478 (78.5)	404 (70.8)	882 (74.7)	
Missing	30 (4.9)	35 (6.1)	65 (5.5)	
Drink Category at Visit 64, n (%)				
None	115 (18.9)	111 (19.4)	226 (19.2)	0.3831
Low/Moderate	368 (60.4)	322 (56.4)	690 (58.5)	
Moderate/Heavy	80 (13.1)	73 (12.8)	153 (13.0)	
Binge	20 (3.3)	29 (5.1)	49 (4.2)	
Missing	26 (4.3)	36 (6.3)	62 (5.3)	
Drink Category at Visit 70, n (%)				
None	126 (20.7)	127 (22.2)	253 (21.4)	0.3675
Low/Moderate	355 (58.3)	304 (53.2)	659 (55.8)	
Moderate/Heavy	80 (13.1)	81 (14.2)	161 (13.6)	
Binge	18 (3.0)	24 (4.2)	42 (3.6)	
Missing	30 (4.9)	35 (6.1)	65 (5.5)	
Smoke Category at Visit 64, n (%)				
Never	213 (35.0)	177 (31.0)	390 (33.1)	0.0064
Former Smoker	311 (51.1)	274 (48.0)	585 (49.6)	
Current Smoker	71 (11.7)	103 (18.0)	174 (14.7)	
Missing	14 (2.3)	17 (3.0)	31 (2.6)	
Smoke Category at Visit 70, n (%)				
Never	214 (35.1)	180 (31.5)	394 (33.4)	0.0013
Former Smoker	325 (53.4)	276 (48.3)	601 (50.9)	
Current Smoker	61 (10.0)	97 (17.0)	158 (13.4)	
Missing	9 (1.5)	18 (3.2)	27 (2.3)	
Comorbidities at Visit 64, n (%)				
Hepatitis C	27 (4.4)	39 (6.8)	66 (5.6)	0.0031
Diabetes	73 (12.0)	86 (15.1)	159 (13.5)	0.0616
Dyslipidemia	422 (69.3)	408 (71.5)	830 (70.3)	0.0093
Liver Disease	1 (0.2)	4 (0.7)	5 (0.4)	0.1464
Kidney Disease	53 (8.7)	158 (27.7)	211 (17.9)	<0.0001
Comorbidities at Visit 70, n (%)				
Hepatitis C	27 (4.4)	39 (6.8)	66 (5.6)	0.0018
Diabetes	80 (13.1)	96 (16.8)	176 (14.9)	0.0400
Dyslipidemia	437 (71.8)	426 (74.6)	863 (73.1)	0.1514
Liver Disease	3 (0.5)	2 (0.4)	5 (0.4)	0.7263
Kidney Disease	76 (12.5)	178 (31.2)	254 (21.5)	<0.0001
BMI Category at Visit 64, n (%)				
Underweight	6 (1.0)	9 (1.6)	15 (1.3)	0.0149
Normal Weight	193 (31.7)	218 (38.2)	411 (34.8)	
Overweight	199 (32.7)	178 (31.2)	377 (31.9)	
Obese	131 (21.5)	88 (15.4)	219 (18.6)	
Missing	80 (13.1)	78 (13.7)	158 (13.4)	
BMI Category at Visit 70, n (%)				
Underweight	8 (1.3)	13 (2.3)	21 (1.8)	0.3571
Normal Weight	191 (31.4)	183 (32.0)	374 (31.7)	
Overweight	187 (30.7)	170 (29.8)	357 (30.3)	
Obese	123 (20.2)	96 (16.8)	219 (18.6)	
Missing	100 (16.4)	109 (19.1)	209 (17.7)	

Markers of self-perception of aging

In our analytic frame, the median score for aging satisfaction was 8 (IQR:7-9) with scores of 8-10 indicating high aging satisfaction, and the median age discrepancy was -12.0 (-19.0, -2.0; Table 1), indicating that most participants felt younger than their chronological age. Neither of these factors was differed significantly by HIV status.

Hypertension transitions

The following numbers of participants (% of total participants) in each mutually exclusive transition patterns were seen after three years of follow-up: 1) 205 (17.4%) no HTN to no HTN; 2) 110 (9.3%) no HTN to HTN; 3) 108 (9.2%) HTN to no HTN; and 4) 757 (64.2%) HTN to HTN. Transition probabilities represent the probability of HTN status at visit 70, given their HTN status at visit 64. The final adjusted model (Table 2) included the primary predictors (age discrepancy and aging satisfaction), HIV status, age, race/ethnicity, education, smoking status, inhaled nitrite use, diabetes, dyslipidemia, and kidney/liver disease. Neither age discrepancy (aOR: 1.13 95% CI: 0.35-3.69) nor low aging satisfaction (aOR: 0.88; 95% CI: 0.31-2.52) were associated with transitioning from no HTN to HTN. PLWH were not at higher odds of transitioning from no HTN to HTN, compared to PLWOH (aOR: 1.38; 95% CI: 0.73-2.60), but had higher odds of transitioning from HTN to no HTN (aOR: 2.60; 95% CI: 1.38-4.87). Participants with diabetes (versus without) had higher odds of remaining with HTN (aOR: 3.69; 95% CI: 1.44-9.45).

**Table 2:** Adjusted Association of Covariates at Visit 64 and Hypertension Transition Patterns HTN, Hypertension; est, estimate; PLWOH, People living without HIV; PLWH, People living with HIV *Statistically significant p<0.05

	No HTN to HTN (vs no HTN to no HTN)	HTN to no HTN (vs no HTN to no HTN)	HTN to HTN (vs no HTN to no HTN)
Age Category			
>= 60	0.86 (0.46-1.62)	1.35 (0.73-2.53)	1.40 (0.90-2.19)
< 60	Referent	Referent	Referent
Age Discrepancy			
Older	1.13 (0.35-3.69)	0.69 (0.25-1.94)	0.74 (0.33-1.66)
Younger	1.56 (0.31-7.88)	0.57 (0.11-2.85)	1.14 (0.36-3.59)
Same	Referent	Referent	Referent
Aging Satisfaction			
Low	0.88 (0.31-2.52)	1.33 (0.53-3.34)	1.08 (0.53-2.21)
Medium	1.13 (0.53-2.40)	0.61 (0.27-1.39)	0.82 (0.47-1.42)
High	Referent	Referent	Referent
Race			
Non-Hispanic Black	1.08 (0.47-2.46)	0.72 (0.31-1.68)	1.15 (0.64-2.07)
Hispanic	0.39 (0.10-1.54)	0.58 (0.18-1.89)	0.60 (0.27-1.35)
Other	no est	no est	no est
Non-Hispanic White	Referent	Referent	Referent
Education			
Less than College	0.87 (0.45-1.69)	0.73 (0.38-1.42)	1.03 (0.65-1.63)
Some College or Higher	Referent	Referent	Referent
HIV Status			
PLWH	1.38 (0.73-2.60)	2.60 (1.38-4.87)*	1.29 (0.82-2.04)
PLWOH	Referent	Referent	Referent
Inhaled Nitrite Use			
Yes	1.08 (0.56-2.10)	0.52 (0.26-1.06)	0.81 (0.50-1.30)
No	Referent	Referent	Referent
Smoke Category			
Former Smoker	0.68 (0.35-1.30)	0.84 (0.44-1.61)	1.00 (0.63-1.58)
Current Smoker	1.23 (0.51-2.97)	1.60 (0.66-3.85)	0.91 (0.47-1.78)
Never	Referent	Referent	Referent
Diabetes			
Yes	0.73 (0.17-3.24)	1.94 (0.60-6.21)	3.69 (1.44-9.45)*
No	Referent	Referent	Referent
Dyslipidemia			
Yes	2.37 (1.25-4.51)*	1.96 (1.03-3.75)*	3.63 (2.32-5.66)*
No	Referent	Referent	Referent
Kidney/Liver Disease			
Yes	0.50 (0.20-1.27)	0.90 (0.41-1.97)	1.09 (0.61-1.96)
No	Referent	Referent	Referent

## Discussion

The prevalence of hypertension increased from 73.1% to 82.6% over three years of follow-up in this sample of MACS participants. Contrary to our hypothesis, aging satisfaction and subjective age were not found to be independently associated with hypertension transition status. Most participants reported high aging satisfaction scores and on average, reported feeling 12 years younger than their chronological age. These findings did not differ based on HIV status.

The prevalence of hypertension among SMM observed in this study is much higher than that reported in prior meta-analyses and when compared with the general population. For example, an analysis of NHANES data between 2015 and 2018 found the prevalence of high blood pressure to be 60.1% among those 45 to 64 years of age, the group most representative of our cohort [23]. This may in part be due to our broad definition of hypertension including participants taking antihypertensive medications who have controlled blood pressure, and to our use of the 2017 American College of Cardiology/American Heart Association (ACC/AHA) definition of hypertension rather than the now outdated Joint National Committee, Seventh Report (JNC-7) definition used in prior studies [17,24,25]. However, the prevalence of hypertension when the JNC-7 definition was used, remained high at 56.3% at visits 64. It was however surprising to see no statistically significant difference in the prevalence of hypertension between PWH and PLWOH [26].

This analysis of a high-quality contemporary study contrasts with other studies exploring an association between SPA and hypertension [10,13], and adds to the argument that SPA may not be associated with hypertension. Even if subjective aging is associated with hypertension, the causal relationship is not clear and in fact, there is evidence that an individual’s burden of comorbidities drives their SPA [27]. Given the silent nature of hypertension, elevated blood pressure may contribute less to a patient’s SPA than other more visible and symptomatic conditions such as heart failure or chronic obstructive pulmonary disease [28]. In our population of SMM a number of other comorbidities, including HIV, depression, and diabetes have previously been shown to be associated with their SPA [17], yet hypertension status in our analysis was not.

This observational study has a number of limitations. First, despite adjusting for covariates, residual confounding cannot be excluded. Second, measures of SPA were self-reported, which is subject to social desirability and other biases even when using the validated Philadelphia Geriatric Center Morale Scale. Third, the majority of participants reported high self-perception of aging, which may have affected our ability to detect a more subtle association between hypertension and aging satisfaction. However, the average age discrepancy and high aging satisfaction scores are typical of other studies in this area [9,29]. Fourth, the large number of men with hypertension in the MACS may have limited power to detect meaningful differences in SPA. Fifth, the participants who enrolled in the MACS are a self-selected sample of men only, so our results may not be representative of other samples of SMM living with or without HIV.

The high burden of hypertension identified in our study suggests a need for intensified blood pressure screening among SMM. Future research could focus on evaluating other easily measured psychosocial cardiovascular risk factors likely to contribute to disparities in SMM health, and strategies to counter these conditions.

## Conclusions

More than four in every five participants in this contemporary study of sexual minority men living with or without HIV had a diagnosis of hypertension regardless of their HIV status which suggests a need for enhanced screening and clinical monitoring in this population. No statistically significant association between self-perceptions of aging and transition of hypertension status was found, which may be related to the asymptomatic nature of hypertension. Identification and investigation of other potential individual-level psychosocial factors is warranted to mitigate hypertension and its cardiovascular sequelae in this population.
